# ‘You run out of hope’: an exploration of low-income parents’ experiences with food insecurity using Photovoice

**DOI:** 10.1017/S1368980021002743

**Published:** 2022-04

**Authors:** Payge Lindow, Irene H Yen, Mingyu Xiao, Cindy W Leung

**Affiliations:** 1 College of Literature, Science, and the Arts, University of Michigan, Ann Arbor, MI, USA; 2 School of Social Sciences, Humanities, and Arts, University of California, Merced, CA, USA; 3 Department of Nutritional Sciences, School of Public Health, University of Michigan, Ann Arbor, MI 48109, USA

**Keywords:** Community-based participatory research, Food insecurity, Food environment, Parents

## Abstract

**Objective:**

Using an adaption of the Photovoice method, this study explored how food insecurity affected parents’ ability to provide food for their family, their strategies for managing household food insecurity and the impact of food insecurity on their well-being.

**Design:**

Parents submitted photos around their families’ experiences with food insecurity. Afterwards, they completed in-depth, semi-structured interviews about their photos. The interviews were transcribed and analysed for thematic content using the constant comparative method.

**Setting:**

San Francisco Bay Area, California, USA.

**Participants:**

Seventeen parents (fourteen mothers and three fathers) were recruited from a broader qualitative study on understanding the experiences of food insecurity in low-income families.

**Results:**

Four themes were identified from the parents’ photos and interviews. First, parents described multiple aspects of their food environment that promoted unhealthy eating behaviours. Second, parents shared strategies they employed to acquire food with limited resources. Third, parents expressed feelings of shame, guilt and distress resulting from their experience of food insecurity. And finally, parents described treating their children to special foods to cultivate a sense of normalcy.

**Conclusions:**

Parents highlighted the external contributors and internal struggles of their experiences of food insecurity. Additional research to understand the experiences of the food-insecure families may help to improve nutrition interventions targeting this structurally vulnerable population.

Over the past several decades, food insecurity has persisted as a critical health problem in the USA. Food insecurity is defined by the US Department of Agriculture as the ‘limited or uncertain availability of nutritionally adequate and safe foods, or the limited or uncertain ability to acquire acceptable food in socially acceptable ways^(1)^’. In 2019, 10·5 % of all US households were food-insecure, though the prevalence is higher among households with children (13·6 %), households with young children (14·5 %) and households headed by a single mother (28·7 %)^(1)^. Previous research has shown that food insecurity is associated with lower nutrient intake^(2)^, lower overall dietary quality^(3)^, higher levels of diabetes and other chronic conditions^(4,5)^ and poorer overall health among adults^(6)^. Food insecurity has also been associated with adverse mental health outcomes in mothers^(7,8)^. While the mechanisms underlying these associations have not been specifically explored, a number of factors have been proposed to explain the observed associations between food insecurity and adverse health outcomes, including poor access to healthy food^(9,10)^, neighbourhood risk factors^(11)^ and the experience of psychological distress^(12)^.

Another mechanism for the associations between food insecurity and physical and mental health outcomes might be through the strategies used by low-income parents to manage and cope with household food insecurity. Some of these strategies may be protective, such as utilising social support networks, whereas other strategies might be harmful, such as experiencing stress and isolation. Qualitative studies can be particularly insightful in understanding one’s lived experiences, as not all behaviours or coping strategies can be measured through survey instruments. However, only a few studies employing qualitative approaches have focused on understanding parents’ or caregivers’ experiences with food insecurity. In one survey of 746 caregivers of children in South Carolina, analysis of open-ended responses revealed that participants changed their food purchases or shifted their food resources, employed cost-saving grocery shopping strategies, changed their meal patterns and adapted their existing resources at home to adjust their household food supply^(13)^. Another mixed-methods study of parents in the United Kingdom revealed that parents reduced the size of their meals or skipped meals when food supplies were running low and shielded their children from the experience of food insecurity by relying on low-cost foods or takeaway meals^(14)^. And finally, results of a qualitative study of low-income mothers who had experienced food insecurity during their own childhoods found that women recalled their parents borrowing food from family members and neighbours, using food stamps and food pantries, stealing and gleaning from the environment, suggesting that some of these experiences had lasting impacts^(15)^.

This study aims to build on existing research using an adaption of the Photovoice methodology to explore parents’ ability to provide food for their family within the context of food insecurity. Photovoice is a method of community-based participatory research that uses visual images to provide insight on the perspectives of individuals through their lived experiences, beliefs and narratives^(16)^. Historically, Photovoice has been used to give agency to specific populations who may have little power over the structural forces that impact their day-to-day lives. The main goals of Photovoice are to (1) enable people to record and reflect their community’s strengths and concerns, (2) to promote critical dialogue and knowledge about important issues through discussion of photographs and (3) to reach policymakers^(16)^. To date, this method has been applied to a variety of research topics at the intersection of public health nutrition and health equity^(17–25)^. In one Photovoice study of African American caregivers from Washington DC, participants discussed the strategies they employed to feed their children the food they wanted them to eat through navigating their local ‘food desert’ and relying on family, friends and food programmes^(21)^. Another Photovoice study of Latina mothers who recently immigrated from Central America found that participants desired higher quality, more equitable food in their neighbourhood, the ability to shape foods provided to their children at school and more education around healthy eating and its alignment with their nutrition knowledge^(22)^. Furthermore, a Photovoice study of twelve minority youth in New York City revealed creative solutions to promote healthier eating behaviours, including more education, better access to and promotion of fresh produce and advocating for healthier foods from the food industry^(26)^.

In the present study, we adapt the Photovoice method to explore how food insecurity affected parents’ ability to provide food for their family, their strategies for managing household food insecurity and the impact of food insecurity on their well-being. Understanding these dynamics can inform improvements for the structure of food assistance programmes and how to increase access to resources in a socially acceptable way.

## Methods

This study took place in the San Francisco Bay Area as a sub-study of a larger qualitative research project on understanding the psychological distress of food insecurity in low-income parents and children^(27)^. In particular, the Photovoice sub-study focused on exploring the challenges that families face in providing food for their children within the context of household food insecurity. Conventionally, Photovoice projects engage the study participants at all stages of research. Adaptations were made to the conventional Photovoice method to facilitate parents’ participation and to complement the emergent themes from the larger qualitative study. In the present study, the researchers formalised the research question, parents submitted their photos and discussed them one-on-one with the study coordinator and the data analysis was conducted by the research team. This study was approved by the University of California, San Francisco Human Research Protection Program.

### Procedures

The Photovoice sub-study was conducted between June 2016 and January 2017. Parents initially completed in-depth semi-structured interviews with the senior author about their family’s experience of food insecurity and the impact of food insecurity on their psychological well-being. Parents were then provided a fact sheet about the Photovoice sub-study and asked about their interest in participation. Those who expressed interest were given a brief one-on-one training of the Photovoice method and asked to take photos of the daily challenges they faced ‘(in providing food for their children during difficult economic times)’. The parents were encouraged to interpret the research question broadly and to use the photos to describe their personal narratives around food insecurity. Although digital cameras were available for the study participants, all parents opted to use their personal smartphones to take and send photos to the study staff throughout a 2-week period. Written informed consent was obtained from all participants for the Photovoice sub-study.

After a 2-week period, each parent completed an in-person 30-min semi-structured interview with the study coordinator to review and discuss their photos in greater detail. Parents were asked to select the photos that were most meaningful to them and to provide more in-depth descriptions of the setting and representation of each photo. Specifically, parents were asked: (1) what made you take the picture?; (2) how does the picture make you feel?; (3) what does the picture illustrate about your day-to-day life, your physical health and/or mental health?; and (4) what do you want lawmakers, advocates or researchers to know about your picture? Parents were compensated $40 for their participation.

### Data analysis

Interviews with the parents were audio-recorded, transcribed and checked for accuracy. Thematic analysis was conducted using an iterative, inductive approach. Initially, a draft codebook was developed after three researchers reviewed all the content from all transcripts and photos. Next, two researchers independently coded the transcripts and photos from the codebook, meeting regularly with the third researcher to revise or refine the initial list of codes until a final codebook was reached. Two researchers then used the final codebook to independently code all transcripts and photos, with discrepancies being resolved by the third researcher. The emergent themes presented in the manuscript follow the key steps for community-based participatory research using Photovoice: (1) selection of photos that accurately represent the community’s needs, (2) identifying emergent themes, issues or theories and (3) contextualising of the photos through the participant’s own words^(16)^.

## Results

A total of seventeen parents (fourteen mothers and three fathers) participated in the Photovoice sub-study. The mean age was 39 years, ranging from 28 to 61 years. Four parents identified as non-Hispanic White, six as Black/African American, three as Hispanic and four as multi-racial, Native Hawaiian/Pacific Islander or American Indian/Alaskan Native. Using the US Department of Agriculture 18-item Household Food Security Survey Module, three parents had low food security and fourteen parents had very low food security.

Four primary themes emerged from the parents’ photos and interviews: (1) food environment promotes unhealthy eating, (2) creative strategies to acquire food with limited resources, (3) psychological distress due to food insecurity and (4) treating their children to special foods to cultivate normalcy.

### Theme 1: Food environment promotes unhealthy eating

**Fig. 1:**
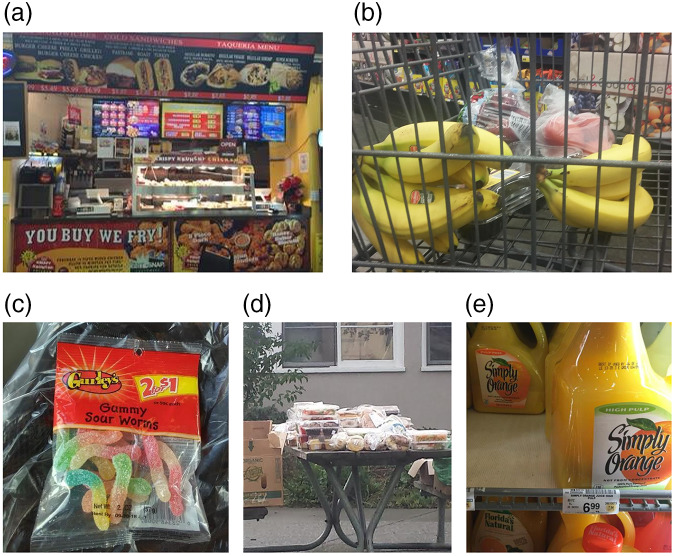
Photos depicting the theme of the food environment promoting unhealthy eating: (a) a fast-food outlet inside a large grocery store, (b) ‘caged’ by the prices of fruits and vegetables, (c) two-for-$1 deal for candy, (d) a pile of baked goods being given away at a food distribution and (e) the high price of orange juice steering parents towards unhealthy alternatives

Parents described multiple aspects of their food environment that promoted unhealthy eating behaviours. Photos were taken from a variety of food outlets (e.g. fast-food restaurant, grocery store, convenience store, local food distribution site) and described the messaging, pricing and excessive availability of unhealthy foods that constantly encouraged unhealthy eating. In Fig. 1(a), the photo depicts a fast-food outlet inside a large grocery store with a promotion for fried chicken. The mother commented, ‘It smells like grease from the fried chicken hut. If you buy chicken from the meat area, they will fry it for you. It’s like ‘come in and get fat,’ you know? Buy this cheap soda. Don’t buy all this other high-quality stuff, because we don’t want you to make a decent quality meal. We want you to eat fried chicken and drink cheap dollar gallon soda’.

Figure 1(b) and (c) illustrates the dichotomy of the high prices of fruits and vegetables compared to the cheap prices of candy. Even foods donated by grocery stores to a local food distribution mostly consisted of sweets and desserts of little nutritional value (Fig. 1(d)). The mother who took the photo said, ‘You can see all the bread and pastry options – filling and delicious – but not healthy. I keep bringing them home, even though I know it isn’t helping my figure one bit’. Another parent described the systemic barriers to healthy eating for low-income families using orange juice as an example (Fig. 1(e)): ‘I think people in poverty are being punished because they can’t afford [orange juice]. They can’t afford healthy food for their kids; they have to give them Kool-Aid and Sunny Delight. That’s sad’.

### Theme *2:* Creative strategies to acquire food with limited resources

**Fig. 2:**
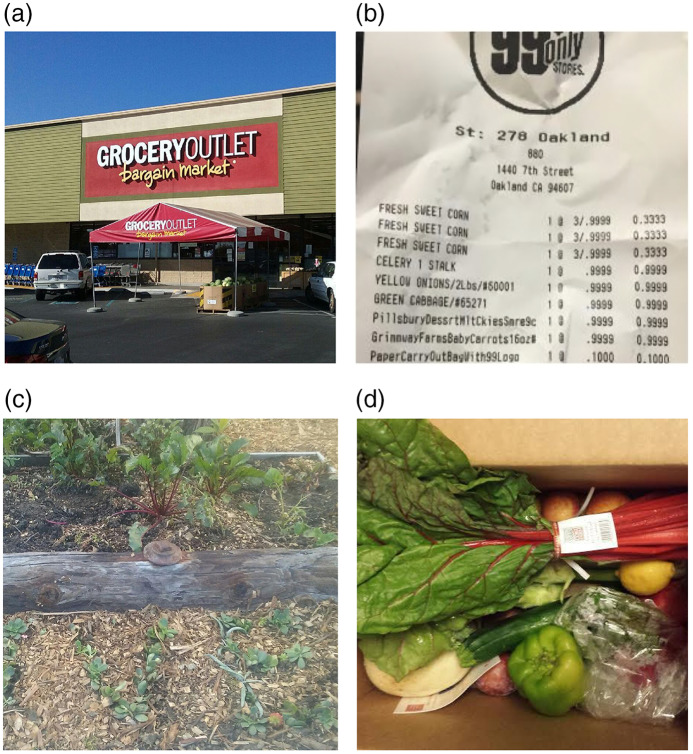
Photos depicting the theme of the creative strategies employed by parents to acquire food with limited resources: (a) one of two Grocery Outlet stores a parent visited that day, (b) shopping for affordable food at the dollar store and (c) an organic garden started by a parent, (d) a produce box from Imperfect Foods

Parents described how they used creative strategies to acquire enough and a variety of food with limited food resources. One strategy was to shop at multiple stores to take advantage of different food promotions and availability. In Fig. 2(a), the parent described shopping at two different Grocery Outlet stores: ‘Every week, they have something different. What you find this week, you may not find next week. I go to two different (Grocery Outlets), so whatever one doesn’t have, usually the other one has’. In addition to low-cost grocery stores, parents frequently shopped at dollar stores for ‘cheap groceries’, including boxed macaroni and cheese and sports drinks (Fig. 2(b)). The mother who took a photo of her receipt said, ‘We didn’t have much money, so hot dogs were a dollar and buns were a dollar at the dollar store. A couple dollar meal’.

Parents were particularly concerned about the need to provide their children with fresh fruits and vegetables. One mother felt it was so important that her children have access to organic foods that she started a garden at her apartment complex (Fig. 2(c)). Another parent subscribed to a weekly Community Supported Agriculture box from Imperfect Foods (Fig. 2(d)), which contained produce that could not be sold due to ‘imperfections or surplus’. She said, ‘even though the produce isn’t perfect, it feeds my family well…. I have to get creative to use something from the box in every meal’.

### Theme 3: Psychological distress due to food insecurity

**Fig. 3:**
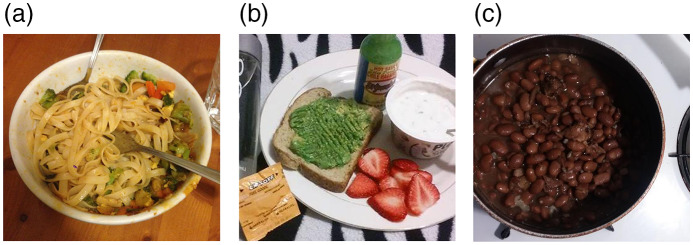
Photos depicting the theme of psychological distress due to food insecurity: (a) Pad Thai from a frozen entrée, (b) a healthy meal after substantial effort which took an emotional toll on the parent and (c) beans and rice to last until the end of the month

Parents expressed feelings of shame, guilt and distress resulting from their experience of food insecurity. These emotions often resulted from having to eat foods that were considered low-quality or cheap, such as ramen noodles, sugary breakfast cereals, hot dogs and frozen meals. As shown in Fig. 3(a), one parent said, ‘Today, I had to eat a frozen pad thai because we didn’t have a good dinner in the house. I really did not like eating this… it made me feel poor’. Even when able to provide a healthy dinner, parents described the amount of effort it took to achieve this goal through budgeting, shopping and meal planning (Fig. 3(b)). The parent said, ‘Eating healthy is really good, but it’s hard to do. I feel like a failure most times because I can never get it right’.

Feelings of guilt were especially magnified when parents were unable to provide their children adequate or nutritious food, knowing that their role was to protect their children from food insecurity. One father showed a photo of his son’s meal of pasta and a breadstick and said, ‘It kills me. It just is killing me. That’s all gone, so he ate that. The only thing I’m concerned about is my son going to be hungry’. Another parent remarked on the difficulty of making it through the month after the initial food resources were depleted and the impact this had on her mental health. She described a photo of beans and rice as: ‘You run out of hope. That’s how it feels right now….the end of the month is like a week and a half [away]. He comes home every day now and says there’s nothing to eat’ (Fig. 3(c)).

### Theme 4: Treating their children to special foods to cultivate normalcy

**Fig. 4:**
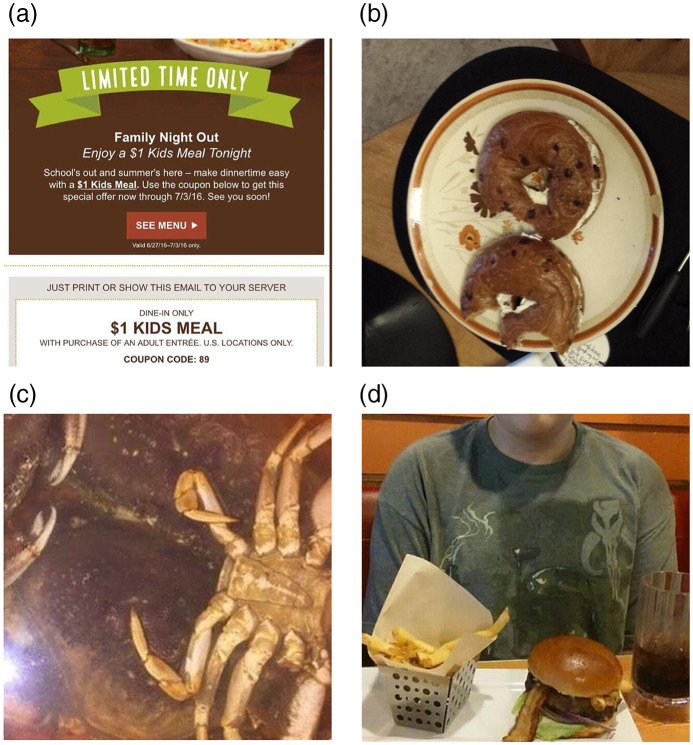
Photos depicting the theme of treating their children to special foods to cultivate normalcy: (a) coupon for a kids meal found on social media, (b) free bagel and cream cheese from a breakfast event, (c) fresh-caught crab for a seafood dinner and (d) participant’s son in front of a burger and fries

Parents described the different ways they treated their children to special foods to cultivate normalcy and shield their children from the stigma of food insecurity. To go out to eat, parents used local restaurant promotions, completed surveys from receipts and found restaurant deals on social media (Fig. 4(a)). One participant, who was a college student, brought her children to campus events with free food. A free breakfast of bagels and cream cheese was a ‘special treat’ (Fig. 4(b)). Another parent took their children crabbing as a way to allow their family to have a nice dinner (Fig. 4(c)). She said, ‘It’s my way of coping. We want seafood, but we can’t buy no $35-pound crab’.

Parents believed it was important for their children to eat desirable foods or dine at a restaurant to promote a sense of normalcy. In keeping their daily routines frugal, they were sometimes able to save up to have a special snack or meal. One mother described treating her son to a Frappuccino as a way to make themselves ‘feel fancy and happy’. In Fig. 4(d), a parent took a photo of her child in front of a typical American meal. She said, ‘Every once in a while, we get the rare opportunity to eat out and my son always picks a burger and fries. (It) doesn’t happen much, but when it does, I feel a sense of normalcy… like maybe we’re not that different from other families’.

## Discussion

Using an adapted method of Photovoice, this study aimed to understand how food insecurity affects parents’ ability to provide food for their family, their strategies for managing food insecurity and the impact of food insecurity on their well-being. Quantitative studies have found associations between food insecurity and poor diet quality^(3,28)^, psychological distress^(12)^ and poor mental health^(29,30)^. The results of the current study help to connect these dots by demonstrating how parents experiencing food insecurity navigate their food environment to feed their children on a limited budget, how the lack of accessibility to and affordability of nutritious foods increased their psychological distress and impacted their perceived perception of their role as a parent and how they attempt to protect their children from the stigma of food insecurity through food rewards and special meals.

In the first theme, parents discussed the multiple pressures to eat unhealthy food placed on them by their food environment. Foods that were unhealthy, highly processed and high in added sugar were not only found in abundance but sometimes aggressively promoted through competitive pricing and signage. Conversely, fruits and vegetables, fruit juice and other healthy items were limited in availability or out of reach due to their high prices. These findings highlight three systemic barriers to healthy eating that co-exist for low-income families: (1) neighbourhood disparities in access to supermarkets and other food stores^(31,32)^, (2) targeted marketing of unhealthful foods and beverages in low-income communities^(33,34)^ and (3) the inverse relation between energetic density and price^(35)^. Parents described these barriers with anger and frustration, demonstrating how the food environment prevented them from making the right nutritional choices for their children despite their desires for their children to eat healthier food.

The second theme identified creative strategies that parents used to maximise their food budget, including shopping at multiple grocery stores and dollar stores. These findings are consistent with prior studies showing how parents and participants in federal food programmes travel farther to shop at their preferred grocery store, use coupons, take advantage of in-store specials and shop at budget stores to stretch their food budget^(13,21,36–39)^. Other strategies like growing their own food and subscribing to a Community Supported Agriculture box demonstrate the importance that food-insecure parents place on fresh fruits and vegetables when feeding their children and are consistent with a prior Photovoice study that equated eating locally with eating healthfully^(24)^.

The third theme describes the psychological burden that food-insecure parents experience while trying to feed their families, including feelings of shame, guilt and distress, providing a potential mechanism to explain why food insecurity has been associated with anxiety disorders and clinical depression in previous studies^(7,40–42)^. As previously documented in a qualitative study of parents with young children^(43)^, the psychological distress resulting from food insecurity was intimately tied to their perceived ability as a parent to provide for their children. Parents are known to sacrifice the quality and quantity of food to ensure their children have enough to eat^(36,44)^. Over time, these behaviours or coping strategies may manifest in poorer mental health for the parent, in part due to their recognition that they cannot fully protect their children from the realities of food insecurity^(43)^.

The fourth theme illustrates how parents aspire to cultivate normalcy for their children through special foods or treats. Through varied approaches, parents found ways to treat their families to restaurant meals, to brand-name splurges and even to luxurious foods such as crab. While another study found that parents use food rewards to celebrate a special occasion or an achievement^(24)^, the present study shows that food-insecure parents use food rewards to protect their children from food insecurity, and perhaps as a coping strategy to relieve their own guilt and distress as parents.

The results of the present study have implications for research and practice. Qualitative studies are useful in providing critical context around the lived experiences of food insecurity. This narrative is important to inform our mechanistic understanding of quantitative studies connecting food insecurity to adverse health outcomes. For example, the present study highlights parents’ psychological distress as a direct result of their experience of food insecurity, which then influenced the foods they provided for their children. While the research on food insecurity and children’s diet and weight status has been inconclusive^(45)^, parental distress may moderate this association and deserves further investigation. Furthermore, these findings can inform the development of community programmes or food assistance policies to better support families with alleviating food insecurity. Programmes that increase neighbourhood healthy food availability or provide financial incentives for fruits and vegetables can eliminate some of the barriers to eating healthfully as described by the parents in the present study. Given the strong influence of food insecurity on mental health, additional resources should also be incorporated into food assistance programmes to more holistically address food insecurity and its sequelae.

The strength of this study is the use of the adapted Photovoice method to give participants a mode of expression for issues that may be best understood through visual and verbal representation. All participants preferred to submit photos using their own mobile phone cameras, which proved to be an efficient and cost-effective method to gather these data. Through the photos submitted and their subsequent interviews, we are able to gain an in-depth understanding of each parent’s unique struggle with food insecurity that could not have been captured through quantitative research methods.

This primary limitation of this study is the adaptations made to the Photovoice method to facilitate participation resulted in low levels of participation across the research process. In the conventional Photovoice method, study participants are highly engaged with researchers in formulating the research question, with discussing and analysing the data as a group, and identifying collective points of advocacy^(16)^, though there is documented variability in participation levels across published Photovoice studies^(46)^. We were not able to build this level of participation into the present study. However, we believe the themes and narratives highlighted from participants’ individual experiences will contribute to the broader policy discussions on improving food security through our federal food programmes and the researchers are actively disseminating the findings from this and the larger qualitative project through professional and public outlets.

This study provides an important narrative to parents’ intimate experiences of food insecurity, ranging from the impact of their food environment, their creative approaches to stretch their food budget, the impact of food insecurity on their psychological well-being and their attempts to use special foods to promote normalcy. Further research should continue to draw on community-based participatory research methods to engage individuals from food-insecure households in identifying focal areas for research and advocacy and to foster systemic change to food access, food equity and food security.
